# Enhancement of plant leaf transpiration with effective use of surface acoustic waves: effect of wave frequency†

**DOI:** 10.1039/c8ra01873a

**Published:** 2018-04-20

**Authors:** Sang Joon Lee, Jeongju Kim, Hyejeong Kim, Jeongeun Ryu

**Affiliations:** Department of Mechanical Engineering, Pohang University of Science and Technology Pohang 37673 South Korea sjlee@postech.ac.kr

## Abstract

Water transport in vascular plants provides remarkable opportunities for various engineering applications due to its highly efficient and powerless transportability. Several previous studies were conducted to regulate the biological responses of plants using noninvasive audible or ultrasound waves. However, the control mechanism of acoustic stimuli applied to plants has not been investigated yet. Thus, the practical application of these stimuli to real plants still exhibits technological limitations. This study experimentally investigated the effects of surface acoustic wave (SAW) frequency on plant transpiration to understand the acoustic-activated leaf transpiration and utilize the advantages of SAW. We captured consecutive images of the enhanced water transport in the test plant (*Epipremnum aureum*) by SAW at three different frequencies (10, 15, and 20 MHz). The dye solution at 15 MHz SAW presented the highest intensity value after 40 min of SAW stimulation. The excitation areas for 15 and 20 MHz SAWs were decreased to 42.3% and 22.6%, respectively, compared with that of 10 MHz SAW. The transpiration rates were directly measured to compare water transport enhancement quantitatively when different SAW frequencies were applied to the same plant leaves. The water transport in the leaves was maximized at 15 MHz SAW, regardless of excitation area.

## Abstract 2

Plant leaf transpiration was enhanced with effective use of SAW activation. The effects on leaf transpiration enhancement were analyzed in detail. The results could be applied to regulate temperature and relative humidity effectively.

## Graphical abstract

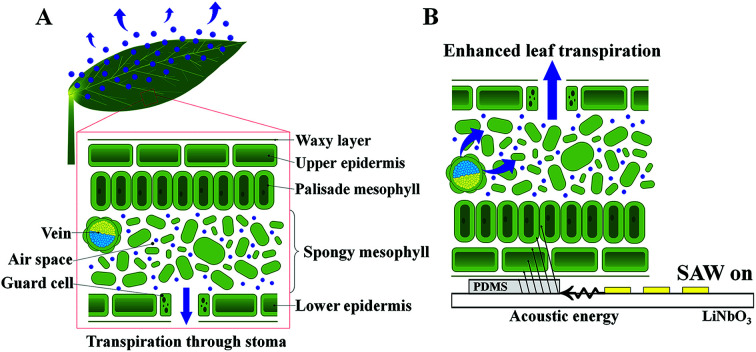

## Introduction

**Fig. 1:**
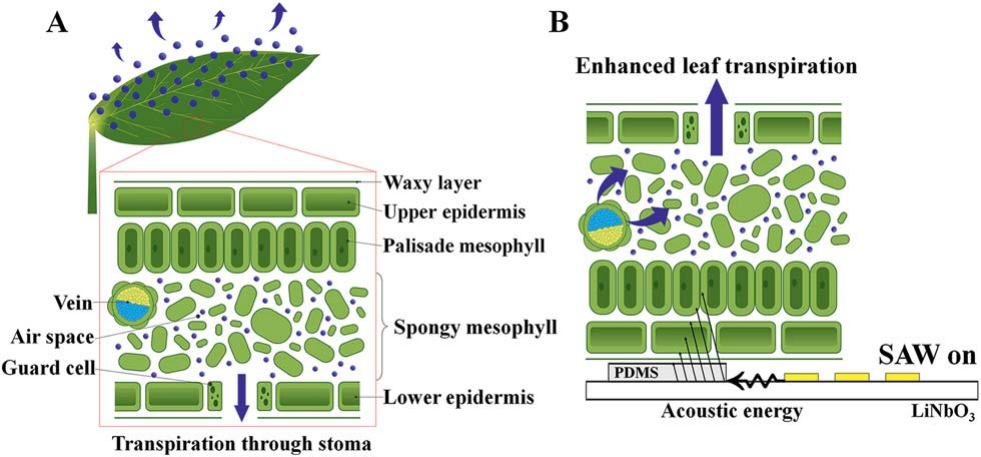
Schematics of the internal structures of a plant leaf and surface acoustic wave (SAW) activation. (A) During plant leaf transpiration, water molecules transported through leaf veins are delivered through the surface of mesophyll cells. Air spaces in the spongy mesophyll cells breathe out water molecules through the stomata. (B) Enhancement of leaf transpiration by using a SAW device. When the acoustic energy is transferred by SAW through the polydimethylsiloxane (PDMS) sheet, water molecules in the air spaces and veins in the spongy mesophyll tissue are disturbed by the acoustic energy. Furthermore, water molecules evaporate quickly through the stomata.

Plant transpiration is the ascending water transport in xylem vessels by evaporating water from aerial parts of leaves (Fig. 1A). The driving force to transport water from roots to leaves is highly negative Laplace pressure, which is generated on the surface of the mesophyll cell wall with nanoscale grooves.^1–3^ Water molecules evaporate and diffuse through the tiny pores of plant leaves, which are called stomata. The opening and closing of stoma are controlled by guard cells to regulate the transpiration rate depending on environmental conditions.^1,4,5^ To diffuse water molecules to the surrounding air, the stomata on the leaf surface should be opened. Plants maintain the balance between water conservation and the uptake of sufficient CO_2_ amounts for photosynthesis. When the stomata are open for photosynthesis, CO_2_ is absorbed into the leaf, and water vapors and O_2_ are discharged.^1,4,5^ Inspired from this plant leaf transpiration, artificial tree and plant towers are installed to remove supersaturated CO_2_, cool down temperature, and control relative humidity.^6–8^ In addition, a leaf-inspired powerless micropump was recently developed.^9^

Several studies were conducted regarding the controls of plants by using audible or ultrasound waves. The effects of sound wave parameters on various plants were investigated at open fields or plantations; these parameters include frequency, sound pressure level, intensity (W m^−2^), exposure period, and distance from the source.^10,11^ Acoustic stimulation causes the stomata to open and increases the water uptake from the ground.^12,13^ Sound waves can also be used to strengthen the immune system of plants. Therefore, previous studies focused on the positive effects of acoustic waves to promote plant growth and organogenesis, increase their production, and consequently improve the product quality.^14–16^ Acoustic waves have also been utilized in microfluidics for precise control of particles, cells, and nanobubbles.^17–19^ Recently, surface acoustic wave (SAW) has been utilized as an active control method to activate plant leaf transpiration. When a plant leaf is exposed to SAW, the intensity of the uptake dye solution is varied, and leaf transpiration is locally enhanced.^20^

The use of SAW devices is one of active flow control technologies used for microfluidics.^21–23^ This method has received considerable attention because it is non-invasive, biocompatible, and easily miniaturized. In addition, the wave generation efficiency of the lithium niobate substrates of SAW devices is higher than other materials, because they have better electromechanical coupling factor. Thus, the generated acoustic waves propagate farther along a material surface.^23,24^ Fabricating the device is also easy because polydimethylsiloxane (PDMS) can be used as a medium.^23^ According to these advantages, SAW has been widely utilized to control and separate particles or droplets inside microfluidic devices, microcentrifugation, jetting, and liquid atomization.^21–23,25,26^

In the present study, to enhance plant transpiration actively, the effects of SAW on water transport in plant leaves were experimentally investigated (Fig. 1B). When acoustic energy was transferred with a SAW device, the water in air spaces and veins of the mesophyll tissues was disturbed by the acoustic energy. This water also quickly evaporated through stomata. Plant transpiration through leaf veins was compared under *in vivo* condition for three different radio frequencies (10, 15, and 20 MHz) of SAW. Depending on SAW frequency, the temperature distributions in the PDMS and the energy conversion efficiency in addition to the leaf transpiration are also varied.^27–29^ The leaf transpiration rates were directly measured by weighting the transpiration-based water loss in the same plant leaves with varying SAW frequency. The relative intensity of the uptake dye solution in the plant leaf was also quantitatively analyzed. Excitation areas were also compared according to SAW frequency to determine the optimum excitation frequency at which the plant transpiration was maximally enhanced. The study was carried out to establish a basic technology to enhance leaf transpiration of vascular plants. In near future, the SAW-induced excitation will be applied to air purification plants to enhance the removal of indoor fine dust.

## Material and methods

### Experimental apparatus

**Fig. 2:**
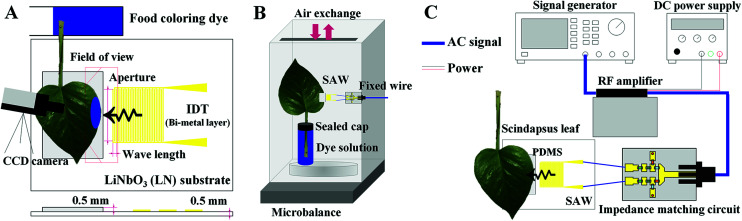
Schematics of the present experimental setups used to evaluate plant leaf transpiration using SAW devices. (A) Edge part of a leaf was cut and dipped into a blue food coloring dye solution and locally stimulated by SAW signals. The plant leaf was stimulated by SAW excitation, and temporal variation of dye solution transported through leaf veins was visualized by using a CCD camera. (B) Experimental setup for measuring leaf transpiration rate using a microbalance. (C) Experimental setup to generate SAWs by using a signal generator, amplifier, and DC power supply. Acoustic waves passed through the self-made impedance matching circuit and transferred to the SAW substrate.

A schematic of the experimental setup is illustrated in Fig. 2C. Sinusoidal electric signals were generated at three frequencies (10, 15, and 20 MHz) with the power of −7 dBm using a signal generator (AFG3021B, Tektronix, USA). The generated signals were amplified with a RF amplifier (LZY-22+, Mini-Circuits, USA) operated by a DC power supply (RDP-303, SMART, Korea). The amplified signals passed through home-made impedance matching circuits (Fig. S1†) and subsequently transferred to the SAW substrates.

### Fabrication of SAW substrates

The SAW substrates were made of lithium niobate (LiNbO_3_, LN, 128° rotated, Y-X, MTI Korea) crystal wafers with 0.5 mm thickness. The interdigital transducer (IDT), which consists of two comb-shaped and interconnected electrodes, was deposited on the LN substrates by using the e-beam evaporation and the lift-off method. To generate the SAW signals effectively, the IDTs were deposited in the order of 5 nm chromium and 70 nm gold. Afterward, the IDTs were deposited with 30 pairs of electrodes with an acoustic aperture of 6 mm. The aperture size should be 10 times larger than the wavelength (*λ*) of SAWs to reduce the diffraction effect.^30^ The IDT for a 10 MHz wave showed intervals of 0.1 mm (*λ*/4). The SAW substrates for three different frequencies were designed with different spacing values according to *λ* = *c*/*f*_SAW_, where *c* is the wave speed of the LN, and *f*_SAW_ is the frequency of the Rayleigh wave.

The PDMS, which was mixed with a curing agent at a ratio of 1 : 10, was prepared with a constant thickness (0.5 mm) using a spin coater (Spin-3000D, Midas System Ltd., Korea) and subsequently cured. To enhance the adhesion of the PDMS, the SiO_2_ layer (200 nm thickness) was used to cover all SAW substrates.^22^ PDMS sheets were attached to the SAW substrates using O_2_ plasma treatment (CUTE, Femto Science, Korea) as a medium between the SAW device and the tested plant leaf.^22^ Given that the LN substrates were vulnerable to heat, they were connected to an impedance matching circuit with a conductive epoxy (CW2400, Chemtronics, USA).

### Measurement of the activated leaf transpiration

The effects of SAW activation on plant leaf transpiration were analyzed by comparing the intensity variations on the SAW-excited plant leaves, which were dipped into a dye solution (Fig. 2A). Leaves of *Scindapsus* (*Epipremnum aureum*), a typical air-cleaning plant, were used as test samples. Side edges of each plant leaf were fixed on the PDMS sheet with the aid of an adhesive tape (Scotch Magic Tape, 3M) to secure reliable adhesion. To make the plant leaf of complete contact with the PDMS, a polystyrene sheet was placed under the SAW substrate, and the plant leaves and the sheet were tightened up using the adhesive tape (Fig. S3†). The tape was attached to the top surface of the plant leaf. Since most stomata are distributed on the bottom surface of plant leaves, the tapes attached at outside the field of view (Fig. 4A) do not have noticeable influence on the transpiration results. The leafstalks about 7 cm in length were cut. They were soaked in an aqueous solution mixed with a food dye (Blue food dye, Wilton), which was diluted to a volume ratio of 1 : 5 using distilled water. Leaf margins, the farthest parts from the midrib of leaves were selected as the excitation region. As the morphological features at the plant leaves are nearly similar, transport phenomena in the microstructures of the leaves could be comparatively observed. The initial volume of the aqueous solution at every experiment was kept at 40 ml in the reservoir.

To visualize the temporal variations of water transport, leaf images were consecutively captured with a CCD camera (RETIGA 4000R, Qimaging, USA) at intervals of 10 min for 2 h. Water evaporation rate through stomata was measured for 12 h using a microbalance (AP250D, OHAUS, USA) (Fig. 2B). The wire for SAW signals was fixed to prevent inadvertent vibrations, which may induce unnecessary experimental errors. The gap between the reservoir containing the dye solution and the plant stalk was tightly sealed to avoid the atmospheric evaporation of water through the gap. The environmental condition was maintained at constant temperature of 25 °C and relative humidity of 40%.

To analyze the effects of temperature increase caused by operating the SAW device on the leaf transpiration, the surface temperatures of the PDMS sheets were measured with a temperature sensor (Vernier LabQuest with a temperature probe) (Fig. S4A†).

## Results

### Excitation of plant leaf transpiration by SAW

**Fig. 3:**
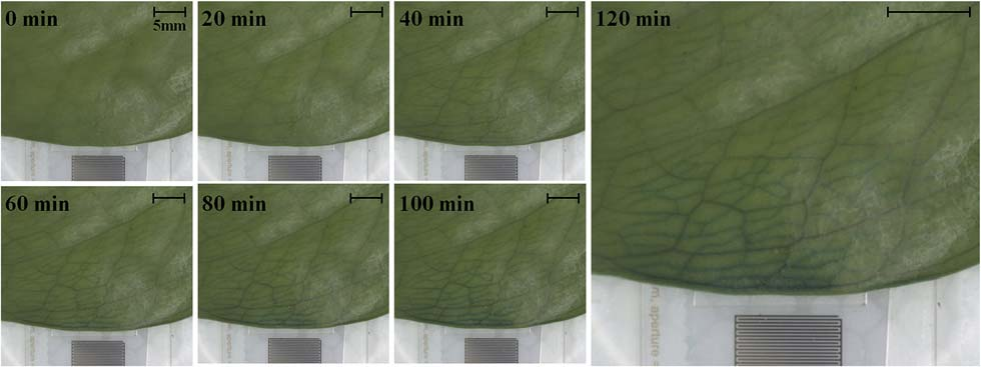
Sequential images showing the local delivery of dye solution in the tested leaf under 10 MHz SAW activation for 120 min with time intervals of 20 min (scale bar: 5 mm).

**Fig. 4:**
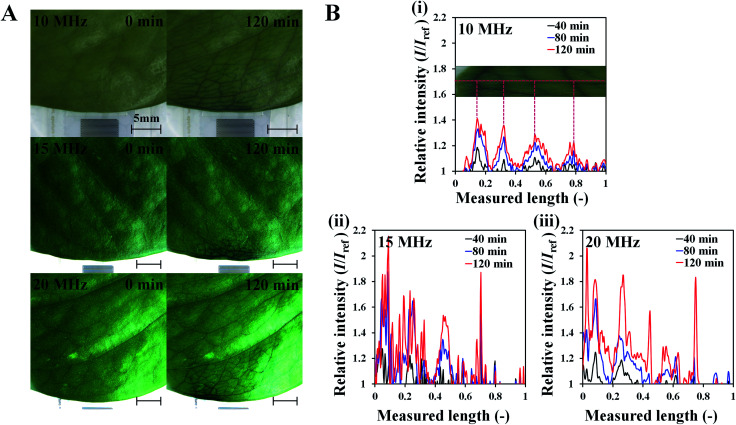
Enhancement of plant transpiration with SAW excitation at various frequencies. (A) Images showing plant leaf transpiration at the initial condition and after 120 min SAW excitation at 10, 15, and 20 MHz (scale bar: 5 mm). (B) Variations in relative intensity (*I*/*I*_ref_) of dye solution according to dimensionless length (measured length = length/total pixel length) at 40, 80, and 120 min SAW excitation at (i) 10, (ii) 15, and (iii) 20 MHz.

To investigate the effects of SAW on plant leaf transpiration, the transport of dye solution through the leaf was visualized for 120 min after applying 10 MHz SAW to a plant leaf (Fig. 3). Afterward, 15 and 20 MHz SAWs were also applied in the same manner (Fig. S2†). The main veins were visibly stained with the dye solution after 20 min of SAW stimuli. In 80 min of SAW stimulus application, the morphological structure of the leaf vein was highly visible due to enhanced dye staining. When the dye solution became darker than those of other parts, it was transported toward downstream branches of small leaf veins. The effects of SAW activation on water transport became remarkably evident with time. Finally, after 120 min, the dye intensity at a local point of the leaf for cases of 15 and 20 MHz SAW stimuli was approximately two times higher than the initial value (Fig. 4B).

### Effects of SAW frequencies on leaf transpiration

To study the effects of SAW frequency on plant leaf transpiration, SAWs (10, 15, and 20 MHz) were continuously applied for 120 min, and the enhanced transpiration was quantitatively analyzed. Fig. 4A shows the optical images of the initial state of test plant leaves and those after 120 min of SAW excitation. Each case exhibited different dye intensities and dissimilar excitation areas. To perform quantitative analysis, the dye solution concentration in the effective area of SAWs was indirectly estimated by measuring the intensity variation in the solution. The intensity variation along an arbitrary same line on the test leaves was acquired at 10 min interval for each experimental condition. Relative intensity value (*I*/*I*_ref_) was defined as the ratio of intensity value (*I*) to the initial intensity value (*I*_ref_). Given that the number of pixel points was divided by the total pixel length, the measured length ranged from 0 to 1 (Fig. 4B). With the continued SAW excitation, the relative intensity gradually increased with time for all cases. The peak intensity values occurred at the main veins where relatively large amount of solution passed through (Fig. 4B(i), red dotted lines). Relative intensity values under 15 and 20 MHz SAW excitations were higher with more complicated fluctuations than those under 10 MHz SAW. This result indicated that the plant leaves evaluated in 15 and 20 MHz SAW excitations may activate more veins than that of 10 MHz SAW. In addition, dye solution may be highly concentrated at mesophyll cells and the veins of the plant leaves.

**Fig. 5:**
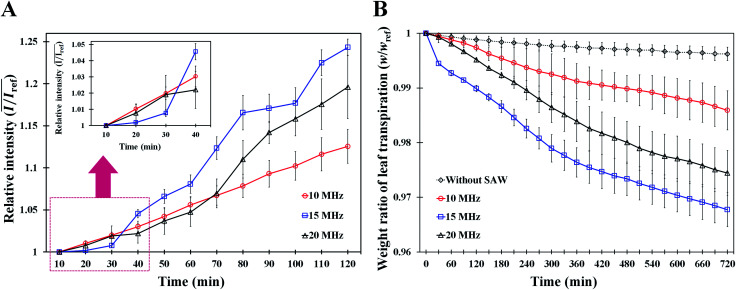
(A) Temporal variations in the relative intensity of dye solution transported through leaf veins over time at three different SAW frequencies (10, 15, and 20 MHz). The intensity values were measured from randomly selected five lines in a single leaf per frequency and they were statistically averaged. (B) Temporal variations in weight decrement ratio of leaf transpiration measured at intervals of 30 min for 12 h with or without SAW activation. Weight loss ratios were obtained from three leaves independently and they were statistically averaged for each frequency.

The average relative intensity value 
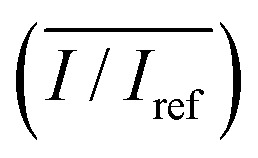
 was estimated by using the data obtained from five randomly selected parallel lines in the excitation area of the leaves (Fig. 5A). The relative intensity increased linearly for all frequency conditions. The average relative intensities for 10 and 20 MHz SAW cases increased in a similar manner in the first 70 min after the start of SAW application. After 70 min, the average relative intensities of 20 MHz SAW rapidly increased to considerably higher values than those of 10 MHz SAW. The relative intensities for 15 MHz SAW were low during the initial 30 min. Their values subsequently increased after 40 min of acoustic activation. The effects of SAW frequency on leaf transpiration rates were directly evaluated by measuring the temporal variation of transpiration-induced weight loss (Fig. 5B). Weights (*w*) of three different leaves for each SAW frequency were measured using a microbalance at intervals of 30 min to monitor the temporal variations of weight loss due to leaf transpiration. All the measured weights were divided by the initial weight (*w*_ref_) to get weight decrement ratios (*w*/*w*_ref_), and then they were statistically averaged. To minimize the biological diversity caused by the use of alive leaves, the weight loss measurements were conducted carefully under the well defined experimental conditions. In addition, plant leaves with similar area and thickness were selected as the test samples to get reliable experimental data. The effects of leaf transpiration were constantly maintained when SAW was not applied. When the SAW device was turned on, the transpiration effect was immediately activated at all SAW frequencies. The largest weight loss by leaf transpiration was observed at 15 MHz SAW. This result is consistent with the maximum SAW frequency for the relative intensity of dye solution. The temporal variations in relative intensity during the first 40 min were comparatively complex without any clear feature compared with the consistently decreasing tendency in weight decrement ratio of maximum SAW frequency for leaf transpiration according to SAW frequency. This result may be attributed to the randomly distributed micromorphological structures of veins in the tested plant leaves.

In general, plant transpiration depends on many environmental parameters, such as temperature, relative humidity, and gas concentration.^1^ Although most of the parameters can be maintained as constant, the acoustic energy delivered from the SAW substrate was transferred through the PDMS in the form of thermal energy. To assess the effects of temperature on plant leaf transpiration, the surface temperature of the PDMS on the SAW substrate was measured for the three frequencies (Fig. S4B†). As an environmental factor, temperature is closely related with metabolism activity of plants. Thus, we carefully monitored temperature variation of the PDMS surface which directly adhered to the plant leaves according to SAW frequency over time. Temperature was measured at the point indicated at Fig. S4A† of the PDMS surface. The contact point of the temperature probe was determined in consideration of the refraction angle (∼16°) where the maximum temperature could appear.

In addition, since the room temperature and the relative humidity was maintained at 25 °C and 40% during the experiments, the heat generated by the PDMS was mostly dissipated into the atmosphere. The PDMS surface was kept at nearly 30.5 °C at the three SAW frequencies (10, 15 and 20 MHz), as illustrated in Fig. S4B.† Each experiment was continued for 30 min. The SAW device was operated at 1 min after starting the temperature measurement. The surface temperature of the device rapidly increased in the first 5 min, reached a nearly saturated state before 10 min, and remained at 30.5 °C ± 0.5 °C with a little sinusoidal variation. The temperature increase caused by the SAW activation may influence the leaf transpiration. However, given that the temperature variation patterns of the substrate at the three different frequencies are similar, the effects of temperature increase may be insignificant under the present experimental environment.

### Effects of SAW frequencies on excitation area

**Fig. 6:**
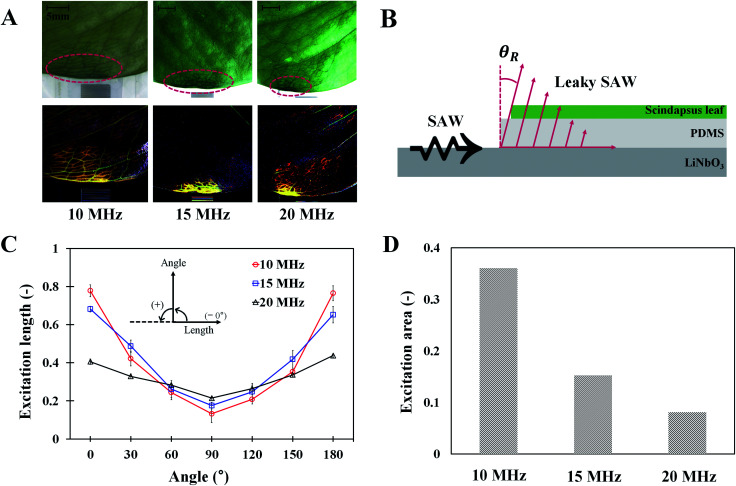
Measurements of the excitation lengths and areas, and comparison of the attenuations of longitudinal component. (A) Optical images of the upper line show the tested leaves at 120 min of SAW stimuli. Each red dotted circle indicates the region where the dye solution delivered. The lower line images show the excited parts where leaf transpiration was enhanced (scale bar: 5 mm). (B) A schematic diagram of leaky SAW attenuating through the PDMS sheet. (C) Variations in excitation length of plant leaves by various SAW frequencies according to measurement angle. (D) Comparison of the excitation areas in plant leaves by various SAW frequencies.

To investigate the effects of SAW frequency on the effective area of leaf transpiration, the excitation lengths and areas on the activated leaves under different SAW frequencies were directly compared (Fig. 6). Only the stained area in the leaves remained and became highlighted by subtracting the optical image acquired after 120 min SAW excitation from the initial optical image (Fig. 6A). The ground effect is negligible, because there is no ground current in the circuit using the LC balun (more detailed information in Fig. S1†). Slight phase shift occurred in the current due to a little mismatched values of LC elements. Due to this phase shift, the phase difference was slightly deviated from 180°. Thus, the left side of the leaf was slightly more influenced at 10 and 20 MHz excitations.

The effects of SAW frequency on the variation in excitation length according to angle are illustrated in Fig. 6C. The effective excitation length was defined as the region where the dye solution was diffused and concentrated in the vein and mesophyll cells. Therefore, the length from the center to the minimum location, where the measured intensity value is zero, was determined (Fig. S5†). The central location used for the analysis was arbitrarily selected at the edge of the leaves where the lengths of 700 total pixels along the 0° and 180° directions were symmetrical. Zero intensity indicated that the dye solution was not diffused into the mesophyll cells. When the measured length was divided by the length of total pixels, the excitation length ranged from 0 to 1. At the measurement angle of 90°, the excitation length for 20 MHz SAW was the longest. When the angle was far from 90°, the excitation lengths at low SAW frequency were increased largely. Notably, the variation curves according to the angle were well fitted into a quadratic function with high *R*^2^ values of 0.991, 0.980, and 0.957, respectively, in the conditions for 10, 15, and 20 MHz SAWs.

The excitation areas in plant leaves at the three SAW frequencies were quantitatively analyzed by comparing their excitation areas (Fig. 6D). Hence, optical images were converted into binary images by utilizing the isodata threshold method;^31^ the number of pixels, where the dye solution was accumulated, was also counted. The excitation area was estimated by dividing the number of counted pixels by the number of total pixels in the region of interest. With the increased SAW frequency, the excitation area decreased rapidly. The excitation areas for 15 and 20 MHz SAWs were 57.7% and 77.4% decreased, respectively, compared with that of 10 MHz SAW.

## Discussion and conclusions

In this study, the effects of SAW frequency on the water transport enhancement capacity of plants by acoustic wave stimuli were experimentally examined. When SAW was not applied to plant leaves, water molecules evaporated from the mesophyll cell tissues of the leaves and underwent regular evaporation activities, thereby emitting the molecules through the stomata. When the SAW device was turned on, the acoustic waves generated by the IDT were converted into acoustic energy at the PDMS sheets; consequently, the transpiration phenomena in the plant leaves were enhanced (Fig. 1B).

The acoustic energy of the SAW device was exerted on the water in veins and mesophyll cells to evaporate water molecules rapidly into the excitation area. When the negative hydrostatic pressure is generated by water evaporation into the air spaces, the water in the plant is driven by the suction pressure and capillary force.^32^ Given that the mesophyll cells can be considered a hydrophilic porous medium, the water transport enhancement by SAW can be explained by water transport or evaporation in micro- and nanoporous channels, such as papers,^32^ threads,^33^ and graphenes.^34^ Therefore, when water molecules inside a plant leaf evaporate, only the dye solution and concentrates remain in the excitation area of the leaf.

The SAW operating time is closely related to the amount of leaf transpiration. Hence, the relative intensity of dye solution increased linearly with the time of SAW operation (Fig. 5A). The solution transport from the stalks of tested leaves was visualized and quantitatively analyzed. The weight loss result according to SAW frequency supported the enhancement effect of SAW activation on leaf transpiration (Fig. 5B). In consideration of the amount of transpiration-based water loss and accumulated solution concentration, the most efficient SAW frequency for activating leaf transpiration was 15 MHz. The temporal variations in relative intensity according to SAW frequency after 60 min of SAW application coincided with those of weight decrement rate in leaf transpiration. This result implied that the variation in relative intensity with SAW application can be effectively used to evaluate the enhancement of leaf transpiration by SAW activation.

The effective areas influenced with SAW activation can be explained by the propagation of Rayleigh waves, which radiate energy when acoustic waves come in contact with a medium surface. Leaky SAWs are generated when acoustic waves propagate with different speeds between the PDMS and LN substrates. The wave propagates into the PDMS with an angle of *θ*_R_ ∼ 16° following Snell's law (Fig. 6B).^27^ Leaky SAWs generated heat energy through the oscillation of PDMS molecules, and the penetration depth (*δ*) had a relationship with 0.7 power of the SAW frequency (*δ* ∼ *f*^−0.7^).^27–29^ The heating in the PDMS layer was rapid and fairly uniform in the excitation area. Acoustothermal heating was generated by viscoelastic damping after acoustic waves were absorbed in viscoelastic materials like PDMS. The excitation length and area are closely related to the attenuation coefficient (*α*) of a leaky wave (*α* ∼ *ρ*_P_*C*_P_/*ρ*_R_*C*_R_*λ*_R_ [m^−1^]), where *ρ*_P_ and *ρ*_R_ are the densities of the PDMS and LN substrates, respectively. *C*_P_ and *C*_R_ represent the wave speeds of the PDMS and LN substrates, respectively, and *λ*_R_ indicates the wavelength.^23^ Following this relation, the attenuation coefficient is inversely proportional to the applied frequency. Thus, with increased SAW frequency, the propagation length of the SAW decreases. This relationship is well illustrated in the experimental results in Fig. 6C at measurement angles of 0° and 180°. However, their relation is difficult to apply into other angles because we ignored the intensities higher than the cut-off value (Fig. S5†), in consideration of the effect of solution concentration in the veins and mesophyll cells. Moreover, with regard to the excitation area, the experimental results agree well with the above relation. The attenuation of acoustic waves in the excitation areas is proportional to the square of length scale and inversely proportional to the square of SAW frequency (∼1/*f*_SAW_^2^). Consequently, the excitation area at 15 MHz (*A*_15 MHz_) is 2.36 times larger than *A*_10 MHz_. In addition, *A*_20 MHz_ is 1.87 times larger than *A*_15 MHz_. The inverse square of the excitation frequencies is 2.25 and 1.78 times, which correspond well with the relation (Fig. 6D).

To enhance plant leaf transpiration with a control modality, we adopted SAW activation to real plant leaves. The effects of SAW operation time and frequency on water transport in plant leaves were experimentally investigated. When the acoustic energy generated by the IDT of the SAW device was delivered through the PDMS sheet to the target area, water evaporation was activated in the plant leaves, and water transport was actively enhanced. To determine the optimum SAW frequency, the effects of three SAW frequencies (10, 15, and 20 MHz) on the water transport and leaf transpiration rate were examined. The variations in relative intensity show that water transport rate increases linearly over time regardless of SAW frequency. The temporal variations in leaf transpiration rate according to SAW frequency are similar to those of relative intensity. The effective excitation length and the excitation area decrease with the increased SAW frequency. In particular, the excitation area is inversely proportional to the square of SAW frequency, and it reaches maximum at 10 MHz SAW due to leaky SAW waves and attenuation coefficient. In consideration of the relative intensity of the accumulated solution and the transpiration-based water loss, the optimum SAW frequency for enhancing leaf transpiration is 15 MHz. Given that plant leaves can be mimicked by microfluidic devices with various porous structures, the present result on SAW frequency effect can be used to enhance water transport in these devices.

Based on the present results, several applications of SAW activation can be considered. In plant hydrodynamic viewpoint, the SAW excitation can be applied to improve plant metabolism, such as growth and photosynthesis of plants, and consequently yield considerable products. SAW can also be utilized to micro- and nanoscale porous structures, as inspired by plant leaves for effective water transport with high efficiency. SAW can be utilized in plant-based engineering application, including plant-covered buildings, green walls, and vertical gardens to regulate building temperature and relative humidity effectively in an energy-saving manner.^7^ In addition, the present results would be applied to various environmental issues, such as enhanced removal of indoor fine dust using air-purifying plants.

## Conflicts of interest

There are no conflicts to declare.

## Supplementary Material

RA-008-C8RA01873A-s001

RA-008-C8RA01873A-s002

RA-008-C8RA01873A-s003

RA-008-C8RA01873A-s004
