# Supplementary material for: Enhancement of plant leaf transpiration with effective use of surface acoustic waves: effect of wave frequency
Source: RSC Adv. 2018 Apr 20;8(27):15141–8. doi: 10.1039/c8ra01873a (PMC9080046; doi:10.1039/c8ra01873a)
Supplement: RA-008-C8RA01873A-s001 [file RA-008-C8RA01873A-s001.pdf]

Electronic Supplementary Material (ESI) for RSC Advances.
This journal is © The Royal Society of Chemistry 2018

Electronic Supplementary Information

Fig. S1. Performance of the fabricated impedance matching circuit. (A) Variation in reflection coefficients with respect to operation frequency and impedance bandwidth at 10% reflection. (B) LC impedance matching sequences on Smith chart.

Fig. S2. Optical images of the local delivery of dye solution under (A) 15 and (B) 20 MHz SAW operations for 120 min with time intervals of 20 min (Scale bar: 5 mm).

Fig. S3. A schematic of the experimental setup in which a test leaf was reliably adhered on the PDMS surface. Four pieces of adhesive tape were used to attach the test leaves and the SAW substrates to the polystyrene sheet. With the aid of the adhesive tape and stickiness of the PDMS, the fixed leaf was well adhered to the PDMS surface. The red dotted box indicates the field of view at Figs. 3, 4A, 6A, and S2 (not to scale).

Fig. S4. (A) A schematic of the temperature measurement setup (not to scale). It shows the contact point to measure temporal temperature variation of the PDMS surface. **(B)** Effects of SAW frequency on temporal variation of the SAW device surface over time under the same experimental condition. Temperatures were fixed within 10 min SAW operation at all frequencies. After 1 min of temperature measurement, the SAW device was turned on. The surface temperatures for all SAW frequencies increased remarkably in the initial 10 min and maintained a saturated value with slight sinusoidal variation.

Fig. S5. Measurement of the excitation length that is approximately 0.086 under the condition of 10 MHz SAW and measurement angle of 90°. The dye solution was concentrated in the leaf veins and mesophyll cell tissues.

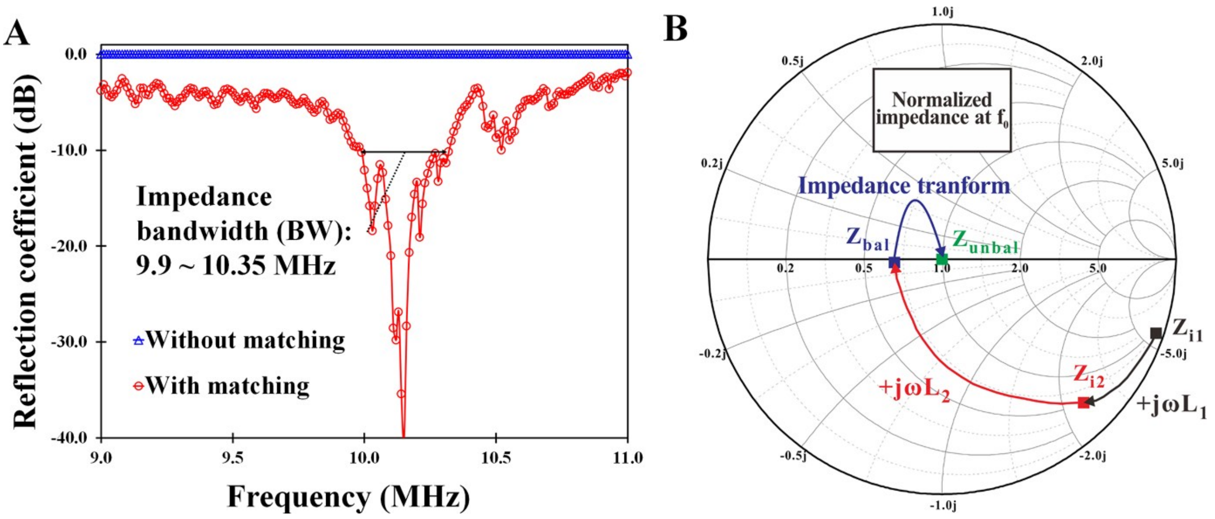

Fig. S1. Performance of the fabricated impedance matching circuit. (A) Variation in reflection coefficients with respect to operation frequency and impedance bandwidth at 10% reflection. (B) LC impedance matching sequences on Smith chart.

The impedance matching circuit which effectively reduces the reflection coefficient was used to get reliable experimental data. The reflection coefficient and the Smith chart for measuring impedance values were obtained by using a vector network analyzer (ZND, Rohde & Schwarz, USA).

To make this experiment effective and reliable, the impedance of the SAW was well matched with the generator. At 10MHz, the measured self-impedance of the fabricated SAW is shown as a black dot in the Smith chart. As illustrated in Fig. S1B, the measured reflection coefficients of the SAW in the frequency range of 9 ~ 11MHz are 0dB (perfect reflection). For impedance matching at the generator frequency of 10MHz, we utilized a lumped-element balun (balanced-to-unbalanced) circuit. Since the balun transforms a purely real impedance into another real impedance, we used serial inductors to eliminate the capacitive component of the SAW input impedance (see black and red traces in Fig. S1B). With the use of the balun, the ground currents were canceled out. As a result, the impedance bandwidth, for which the measured reflection coefficient was less than -10dB, in the range of 9.9 ~ 10.35MHz was achieved (see Fig S1A).

The present result was obtained at 10 MHz impedance matching condition. The other two SAW frequencies (15 and 20 MHz) were also well matched in the same manner.

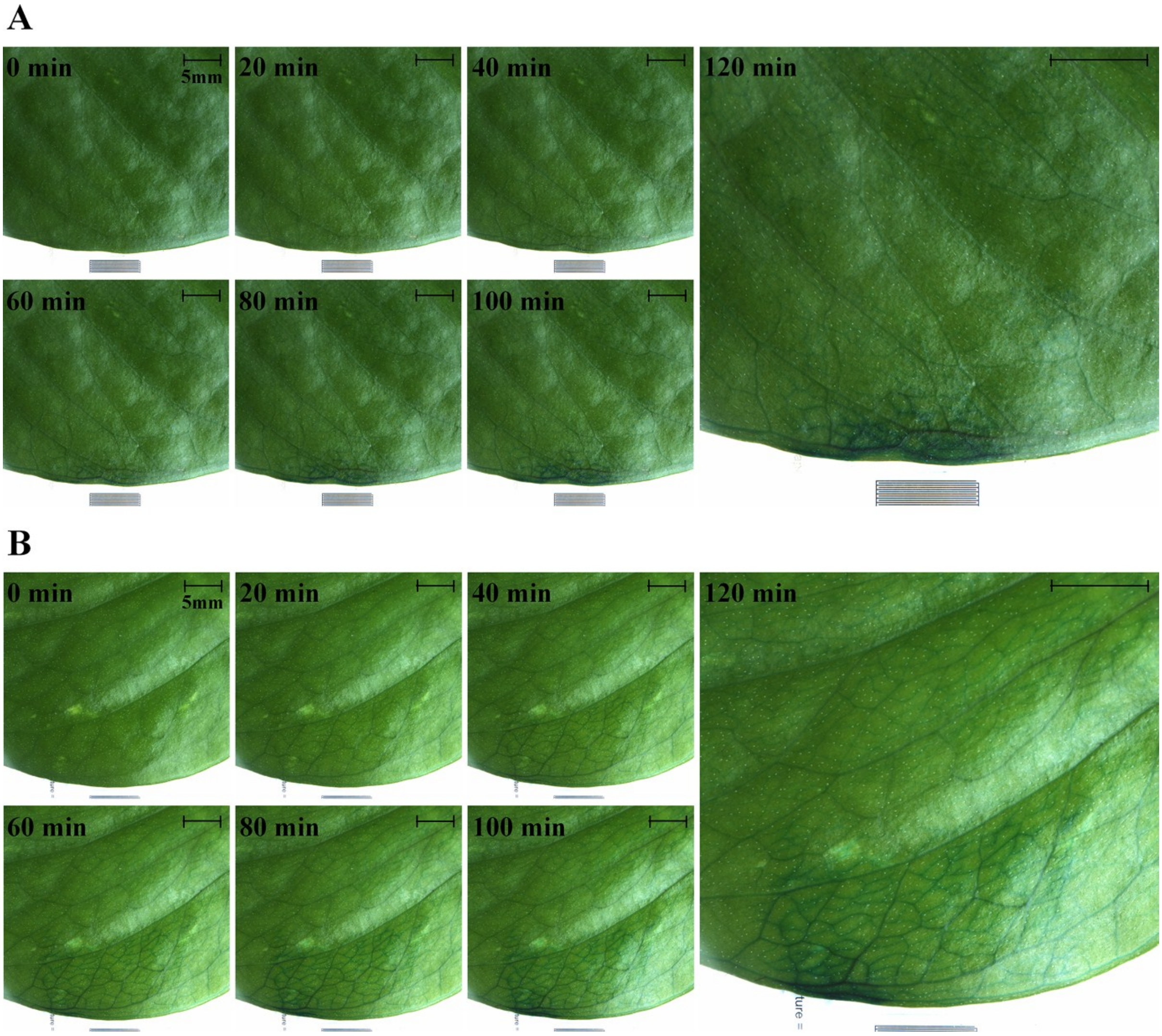

Fig. S2. Optical images of the local delivery of dye solution under (A) 15 and (B) 20 MHz SAW operations for 120 min with time intervals of 20 min (Scale bar: 5 mm).

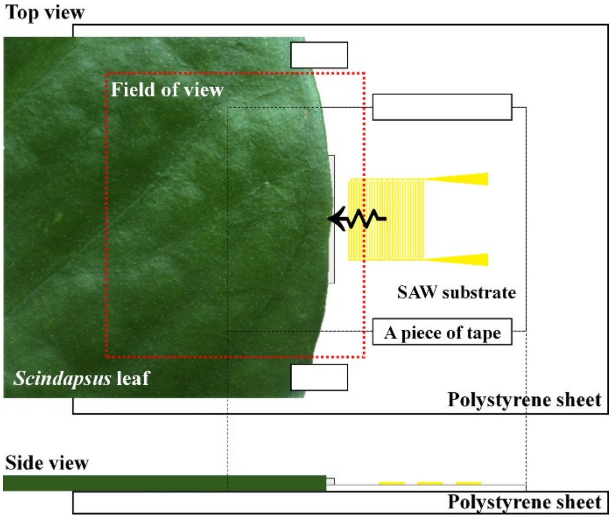

Fig. S3. A schematic of the experimental setup in which a test leaf was reliably adhered on the PDMS surface. Four pieces of adhesive tape were used to attach the test leaves and the SAW substrates to the polystyrene sheet. With the aid of the adhesive tape and stickiness of the PDMS, the fixed leaf was well adhered to the PDMS surface. The red dotted box indicates the field of view at Figs. 3, 4A, 6A, and S2 (not to scale).

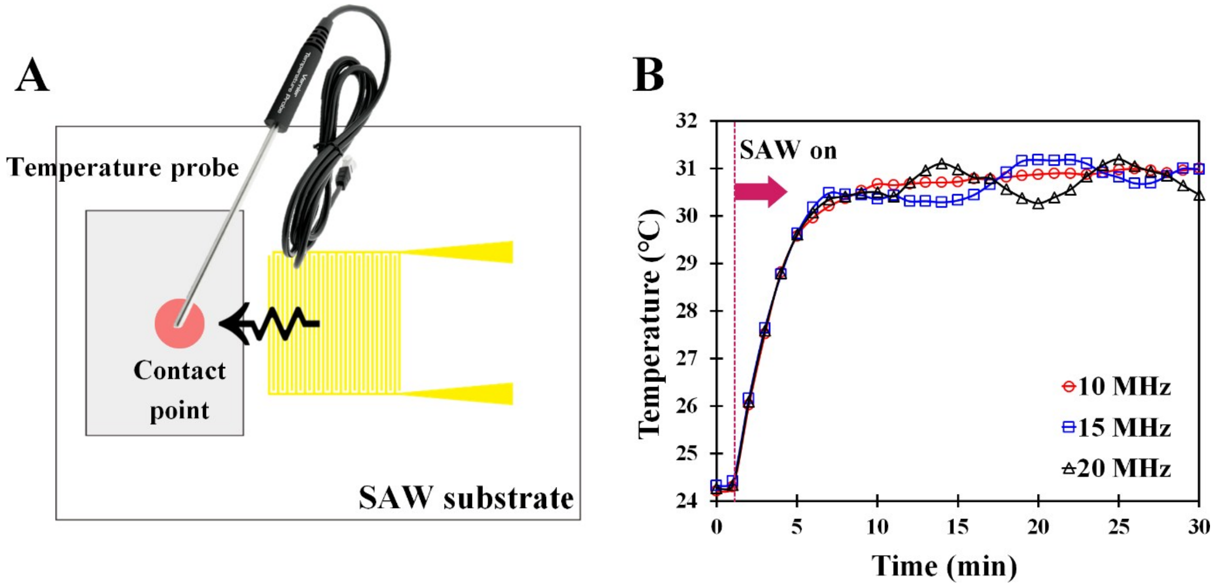

Fig. S4. (A) A schematic of the temperature measurement setup (not to scale). Temporal variation of temperature at the PDMS surface was measured at the contact point. **(B)** Effects of SAW frequency on temporal variation of the SAW device surface over time under the same experimental condition. Temperatures were fixed within 10 min SAW operation at all frequencies. After 1 min of temperature measurement, the SAW device was turned on. The surface temperatures for all SAW frequencies increased remarkably in the initial 10 min and maintained a saturated value with slight sinusoidal variation.

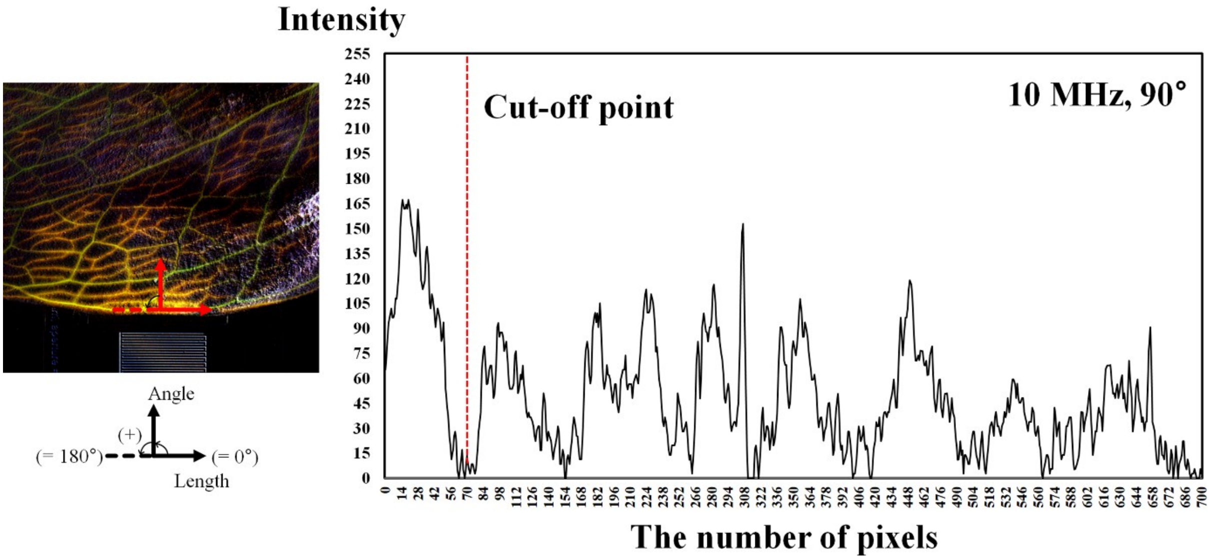

Fig. S5. Measurement of the excitation length that is approximately 0.086 under the condition of 10 MHz SAW and measurement angle of 90°. The dye solution was concentrated in the leaf veins and mesophyll cell tissues.
